# Cloud fraction at the ARM SGP site: reducing uncertainty with self-organizing maps

**DOI:** 10.1007/s00704-015-1384-3

**Published:** 2015-02-15

**Authors:** Aaron D. Kennedy, Xiquan Dong, Baike Xi

**Affiliations:** Department of Atmospheric Sciences, University of North Dakota, 4149 University Ave., Box 31 9006, Grand Forks, ND 58202-9006 USA

## Abstract

Instrument downtime leads to uncertainty in the monthly and annual record of cloud fraction (CF), making it difficult to perform time series analyses of cloud properties and perform detailed evaluations of model simulations. As cloud occurrence is partially controlled by the large-scale atmospheric environment, this knowledge is used to reduce uncertainties in the instrument record. Synoptic patterns diagnosed from the North American Regional Reanalysis (NARR) during the period 1997–2010 are classified using a competitive neural network known as the self-organizing map (SOM). The classified synoptic states are then compared to the Atmospheric Radiation Measurement (ARM) Southern Great Plains (SGP) instrument record to determine the expected CF. A number of SOMs are tested to understand how the number of classes and the period of classifications impact the relationship between classified states and CFs. Bootstrapping is utilized to quantify the uncertainty of the instrument record when statistical information from the SOM is included. Although all SOMs significantly reduce the uncertainty of the CF record calculated in Kennedy et al. (Theor Appl Climatol 115:91–105, 2014), SOMs with a large number of classes and separated by month are required to produce the lowest uncertainty and best agreement with the annual cycle of CF. This result may be due to a manifestation of seasonally dependent biases in NARR. With use of the SOMs, the average uncertainty in monthly CF is reduced in half from the values calculated in Kennedy et al. (Theor Appl Climatol 115:91–105, 2014).

## Introduction

In the past two decades, the Atmospheric Radiation Measurement (ARM) Program has installed and operated a number of remote sensing instruments dedicated to observing cloud macro- and micro-physical properties. These include millimeter-wavelength cloud radars (MMCRs; Moran et al. 1998), micro-pulse lidars (MPLs; Spinhirne 1993), and laser ceilometers. At the most basic level, these instruments measure total cloud fraction (CF), the ratio of the number of vertical profiles with clouds present to the total number of profiles having instrument samples. This quantity can be broken down further to specific layers or cloud types based on height and thickness.

The gross properties of CF at the Atmospheric Radiation Measurement Southern Great Plains (ARM SGP) site have been studied by a number of individuals including Dong et al. (2006), Kollias et al. (2007), Xi et al. (2009), and Kennedy et al. (2010). Most recently, Kennedy et al. (2014) studied the ramifications of instrument downtime and sampling selection on the calculation of total CF at monthly intervals. As downtime increases, uncertainty in monthly CF increases nonlinearly. While ARM has an excellent track record with instrument uptime, Kennedy et al. (2014) found larger uncertainties in monthly CF at the ARM SGP site from 1997 to 2003 due to MPL downtime.

On a first order, cloud occurrence should be associated with the large-scale atmospheric state. With knowledge of what cloud properties are observed for specific synoptic patterns, it should be possible to better constrain ARM observations during periods of instrument downtime. Clouds and meteorological regimes have been linked to each other in numerous studies. Tselioudis and Jakob (2002), Jakob and Tselioudis (2003), Rossow et al. (2005), and Del Genio et al. (2005) have all successfully partitioned International Satellite Cloud Climatology Project (ISCCP; Rossow and Schiffer 1999) cloud regimes by meteorological conditions. Other studies such as Marchand et al. (2009) and Evans et al. (2012) have used self-organizing maps (SOMs; Kohonen 2001) to classify synoptic patterns and link these states to hydrometeor profiles from ARM MMCRs.

The primary purpose of this paper is to better constrain monthly estimates of total CF from 1997 to 2010 at the ARM SGP site found in Kennedy et al. (2014). Missing observations due to instrument downtime will be replaced with statistical information derived from the classification of synoptic states using SOMs. The SOM technique, like most clustering algorithms, requires user input to determine the number of classes and sampling used. This subjectivity is often glossed over. A secondary goal of this paper is to explore the impact SOM class selection and sampling have on synoptic pattern classifications.

This paper is structured as follows: a brief background covering the ground observations of CF and SOMs is provided in “Section 2.” The implementation and classification procedure of SOMs is covered within the methodology (“Section 3”), and two examples of SOMs are shown in “Section 4.” Results of the various SOMs are provided in “Section 5,” and the improved record of CF is given in “Section 6.” The paper concludes with a summary of key findings and future avenues of research in “Section 7.”

## Background

### Ground observations of CF

Cloud observations at the ARM SGP site come from the Active Remote Sensing of Clouds (ARSCL) Value-Added Product (VAP; Clothiaux et al. 2000, 2001). For a complete discussion of how this dataset was processed, the reader is referred to Kennedy et al. (2014). In summary, only observations from the MMCR and combined MMCR and MPL instruments (when both are operational) are considered. Total CF is defined as the ratio of the number of vertical profiles with cloud present to the total number of profiles available. This temporal calculation of CF at the ARM SGP site compares well to area-averaged CFs provided by Geostationary Operational Environmental Satellite (GOES) observations (Kennedy et al. 2010; Xi et al. 2009).

**Fig. 1:**
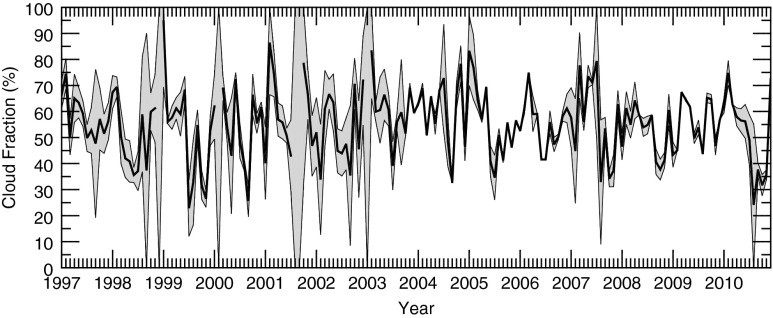
Monthly MMCR + MPL total cloud fraction from 1997 to 2010. 95 % confidence intervals are given by the *shaded grey boundaries*. Figure is adapted from Fig. 5 of Kennedy et al. (2014)

Monthly uncertainties of total CF were calculated using a bootstrap technique for months with instrument uptimes ≥95 %. For these months, samples were randomly withheld in increasing quantities to determine the 95 % confidence interval for a specific instrument uptime. The result of this procedure for the combined MMCR + MPL observations is shown in Fig. 1. The uncertainty varies markedly by month due to varying instrument uptimes (see Fig. 3 of Kennedy et al. (2014)).

### Self-organizing maps

SOMs are an unsupervised competitive neural network that can classify datasets with any arbitrary amount of dimensions (Kohonen 1989). The unique feature of this clustering procedure is the use of a neighborhood function that relates classes to each other in a 2-D matrix known as the feature map. This function gives the SOM a number of advantages over other clustering techniques. Hewitson and Crane (2002) discuss many of these properties and how they are advantageous to synoptic meteorology. In brief, SOMs capture nonlinearities in the data, provide a visually intuitive way to interpret results, and fill in voids within the data space. For example, if a SOM is used to classify time periods of strong and weak low-/high-pressure systems, the feature map will include classes that fall between these two extremes (i.e., Fig. 1 in Hewitson and Crane 2002). Without the neighborhood function, the SOM is reduced to *k*-means clustering (Kohonen 2001).

SOMs have been employed at the ARM SGP site in the past by Marchand et al. (2006, 2009). In these studies, statistically different hydrometeor profiles were characterized for a number of states. Overall, however, these studies sought to limit the number of defined classes. As a result, a limited number of classes occurred for certain seasons such as summer. This may or may not be sufficient to adequately relate the occurrence of CF to the atmospheric state. If the number of classes available for investigating independent variables is too few, excessive averaging will occur. For the case of only one synoptic state being available for a season or month, all cloudy, clear, and partial cloudy scenes will be grouped together, causing the independently determined CF to equal the climatological value. Ample classes are needed for a given period of time to delineate between the varieties of sky conditions.

## Methodology

It is hypothesized that a large quantity of classes must be chosen to capture the variability in CF with the atmospheric state. To test this hypothesis, a number of SOMs were developed with a varying amount of classes. SOMs were developed not only from atmospheric states grouped together for the entire period (1997–2010) but also by month. In the latter case, results from the 12 monthly SOMs were used together to constrain observations for the year. This eliminates the possibility of a SOM selectively choosing more classes during one season than another. SOMs are denoted by the time period (M for monthly and A for annual) and the number of classes. For example, M32 refers to a collection of 32 monthly class SOMs. A full list of the SOMs developed is given in Table 1. Annual SOMs ranged from 12 to 1200 classes while monthly collections of SOMs varied from 32 to 96 classes, providing an effective 396–1152 (12*n) classes for the year. The upper limit of classes was chosen such that all classes had observational data. Larger SOMs yielded select atmospheric states with no MMCR + MPL observations to determine cloud statistics. Common among all of these SOMs were rectangular feature maps to aid the learning process (Kohonen 2001), and the exact dimensions used in this study are provided in Table 1.

**Table 1:** SOMs developed for this study. *A* refers to SOMs conducted over the entire year, while *M* is a collection of 12 monthly SOMs. The effective number of classes for monthly SOMs is given in the rightmost column

Name	Classes	*X*	*Y*	Effective classes
A12	12	6	2	–
A32	32	8	4	–
A60	60	10	6	–
A96	96	12	8	–
A140	140	14	10	–
A192	192	16	12	–
A252	252	18	14	–
A320	320	20	16	–
A396	396	22	18	–
A780	780	30	26	–
A1200	1200	40	30	–
M32	32	8	4	384
M60	60	10	6	720
M96	96	12	8	1152

To capture the gross properties surrounding the ARM SGP site, atmospheric properties were averaged to a 7 × 7, 2.5° × 2° longitude by latitude grid centered on the ARM SGP central facility in a fashion similar to that of Marchand et al. (2006, 2009). Variables input into the SOMs included the mean sea-level pressure (MSLP) and relative humidity (RH), geopotential height (*Θ*), zonal wind (*U*), and meridional wind (*V*) at 900-, 700-, 500-, and 300-hPa levels. Whereas Rapid Update Cycle (RUC; Benjamin et al. 2004) analyses were used in the former studies, a longer term dataset was required for this work. To satisfy this requirement, the North American Regional Reanalysis (NARR; Mesinger et al. 2006) was utilized. Kennedy et al. (2011) has shown that this reanalysis compares well with observed sounding profiles at the ARM SGP site, although some seasonally dependent biases exist (i.e., upper tropospheric RH).

SOMs were trained from ~40,000, 3-hourly NARR samples available over the 14 years. The 3-D arrays of NARR variables were normalized to identical ranges to provide equal weight to the SOM analysis. Without this normalization, SOMs were heavily weighted towards the upper-air pattern due to the large values of geopotential height on lower pressure surfaces. After the normalization, the 3-D fields were decomposed into input vectors with 833 elements (49 elements × 17 variables). These vectors were then used to train the SOMs for the desired period of time.

The training process for SOMs is a two-stage procedure. In the first stage, the SOM uses a relatively low number of training samples (i.e., the number of available 3-hourly synoptic states) with a large learning rate and neighborhood radius to quickly orient the SOM to the data. In the second stage, the SOM converges to a final solution by reiterating over the same training samples multiple times but using a smaller learning rate and neighborhood radius. SOMs within this study used learning rates of 0.05 and 0.01 for stages 1 and 2, respectively. Neighborhood radii in the first stage varied with the maximum dimension of the SOM (*X*_dim_-1). In the second stage, the radii were reduced to one to two nodes depending on the size of the SOM. A total of 100*N iterations were performed to converge to final solutions.

The freely available SOM_PAK software package (Kohonen et al. 1996) was used to perform the training. Using this package, each SOM was randomly initialized 10 times, and the SOM with the smallest map error was saved. Error variance was small (<0.01 %) for the 10 samples regardless of the SOM size, suggesting this was an adequate number of tests. A number of sensitivity tests were performed by varying the neighborhood radii, the learning rates, and the number of second-stage iterations. Changes in the former two had negligible impact on the final error given that an adequate number of iterations were performed (such as 100*N for the second stage).

The result of the SOM training process is an output vector for each class within the SOM. These vectors were then reconstructed into the 7 × 7, 2.5° × 2° array of 17 variables representing the atmospheric state. For every 3-h period from 1997 to 2010, the observed atmospheric state from NARR was compared to the SOM classes. For each time period, the class with the minimum Euclidean distance was chosen as the classified atmospheric state. When instruments were operational, 3-hourly total CFs were calculated to determine the expected CF for each class in the SOM.

## Examples of two SOMs

### Properties of the feature maps

**Fig. 2:**
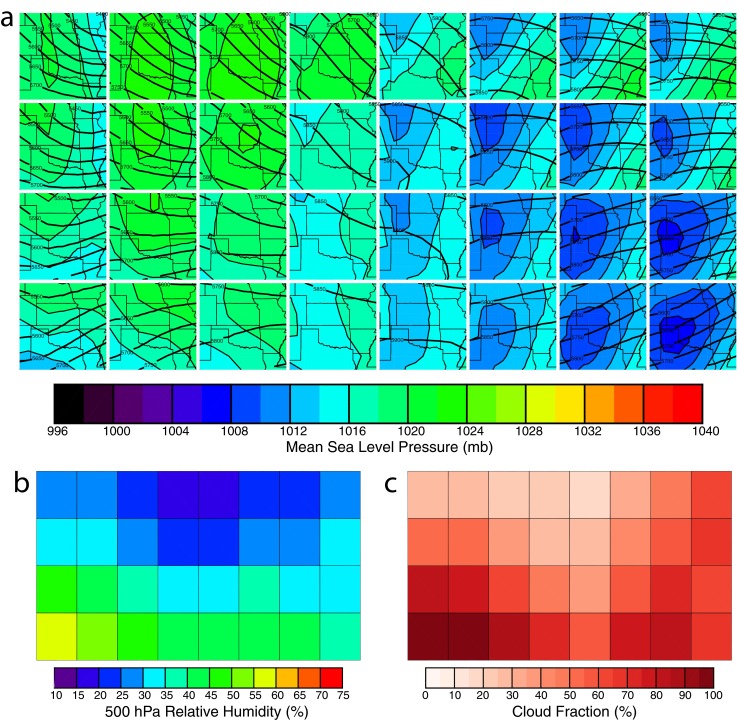
Example 8 × 4, 32-class SOM (A32). **a** 500-hPa geopotential height (*thick contours*) and MSLP (*shaded*). **b** Class mean 500-hPA RH for the point over the ARM SGP site. **c** Class mean total cloud fraction as observed by the MMCR and MPL

To demonstrate common characteristics of an SOM used for synoptic classification, the properties of A32 are presented (Fig. 2). Surface lows associated with troughing at 500 hPa are found in the lower right-hand corner, while surface highs occurring to the west of trough axes are located in the upper-left (Fig. 2a). Depending on the initialization of the SOM, these patterns can orient themselves in a variety of ways; however, high/low pressure in opposing corners is a common result (for another example, see Fig. 2 of Hewitson and Crane 2002). Other orientations include similar synoptic patterns aligned with a diagonal or on opposing sides.

Spatial patterns of individual variables vary gradually across the SOM feature map due to the use of the neighborhood function (Fig. 2b). For the A32 SOM, 500-hPa RH is at a maximum for weak short wave troughs (lower left), while it is lowest for cases of ridging and surface high pressure (upper center). Inspection of the RH fields found that the 2-D fields were consistent with the height and MSLP patterns (not shown). Classes with mid-latitude cyclones had higher humidity at 900 hPa in the warm sector, maximized near the warm front. That said, humidity was lower than what might be expected. With only 32 classes, the SOM must make compromises to span the entire range of atmospheric states over the course of 14 years.

Class mean total CFs calculated using MMCR + MPL observations are shown in Fig. 2c and closely resemble the feature map for 500-hPa RH, indicating the relationship between cloud occurrence and the large-scale synoptic state. Correlations of CF with RH ranged from 0.71 to 0.92 depending on the level chosen. Other than RH, CF had strong correlations with the mid- to upper-level meridional wind, signifying the link between cloud occurrence and large-scale moisture transport. In summary, the A32 SOM shows that CF is higher when troughs exist over the ARM SGP site—an expected result.

**Fig. 3:**
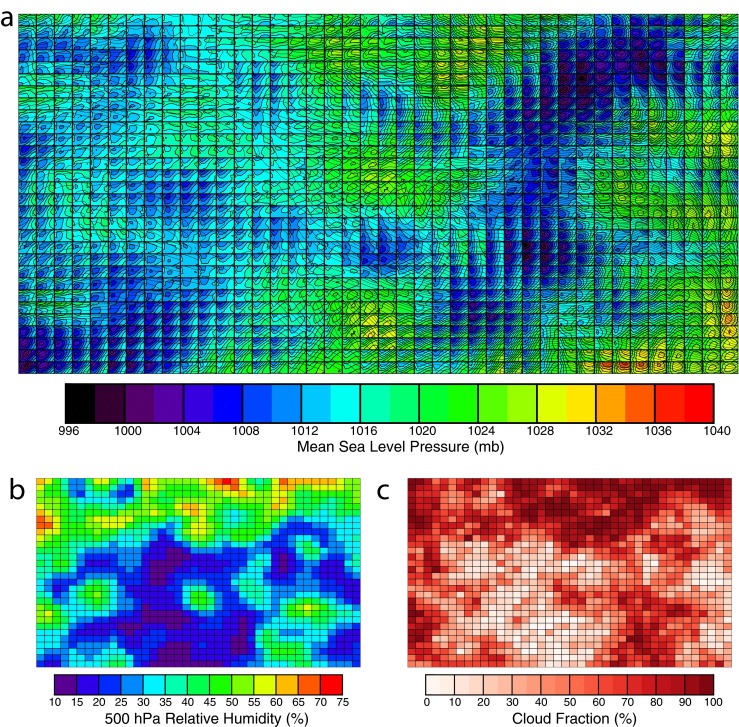
**a**–**c** As in Fig. 2, but for a 40 × 30, 1200 class SOM (A1200). *Color bars* are identical to those in Fig. 2

As SOMs increase in size, synoptic patterns orient themselves in more complicated manners such as those found in A1200 (Fig. 3). Unlike smaller SOMs, high-/low-pressure regimes are found in distinct clusters (i.e., strong low pressure in the upper right and lower left, Fig. 3a). With more classes, less averaging occurs, and the range of values for atmospheric variables increases. For example, from A32 to A1200, MSLP range increases from 1006–1022 to 996–1038 hPa. Another feature of larger SOMs is greater variability for classes with similar properties in specific fields. Many of the classes that have comparable 500-hPa height/MSLP patterns straddle regions with strong gradients in 500-hPa RH (Fig. 3b).

Increasing classes also impacts the characteristics of independent variables such as CF (Fig. 3c). Unlike A32, a number of classes have perfectly cloudy or clear conditions, representing situations such as frontal zones or large-scale subsidence when sky conditions are persistent for hours at a time. Fractional CF is still allowed, and these classes represent either time periods when broken skies prevail (such as summertime cumulus) or time periods when the SOM cannot capture the local-scale forcing for clouds (i.e., mesoscale boundaries). In the latter case, some instances of fractional cloudiness could be mitigated with a higher resolution SOM. This is dependent on the quality of the reanalysis; however, at smaller scales, accuracy is questionable due to the spacing of observations and physical parameterizations used.

Increasing the number of nodes in SOMs raises two potential problems, both demonstrated in Fig. 3. First, independent samples are spread out among more classes. From A32 to A1200, the average number of hours of cloud observations per class decreases from 2945 to 79 h. Variability in class mean CFs is raised across the feature map, slightly decreasing correlations with RH and meridional wind. Whether this hinders the goals of this study will be important to investigate. Second, by the shear nature of a large SOM, one of its main advantages is lost: the ability to quickly visualize relationships between dependent and independent variables. Diagnosing why a certain class mean CF is associated with a particular pattern becomes an exercise in futility.

### Temporal properties of annual SOMs

**Fig. 4:**
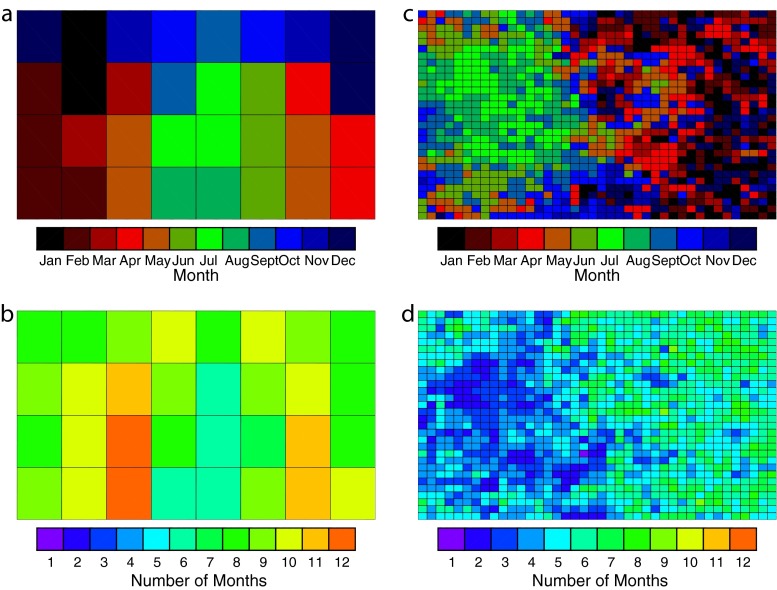
Class characteristics for select SOMs: **a** month of most frequent occurrence and **b** number of months of occurrence for the A32 SOM. **c**, **d** As in **a**, **b** except for the A1200 SOM

Annual SOMs can be used to determine when atmospheric states typically occur. In Fig. 4, two characteristics are plotted for the A32 and A1200 SOMs: (1) the month of most frequent occurrence for each class and (2) the total number of months classes occurred in. More active patterns in A32 (i.e., troughing) occur most often during the late winter and spring months (Fig. 4a) and can occur during the majority of the year (8+ months, Fig. 4b). Summer regimes are found in the center of the A32 SOM and are associated with weak gradients in the 500-hPa geopotential height as the jet stream has moved north of the ARM SGP site (Fig. 4a). Matching the results of Marchand et al. (2009), summer regimes occur less often and are found only during 5–7 months of the year (Fig. 4b).

Similar characteristics are found for the A1200 SOM. Like A32, this SOM also has preferred regions for regime occurrence, with nearly all of the summer classes occurring on the left-hand side of the SOM. Winter regimes are located near the edges and on the right-hand side, with spring/fall classes generally separating the opposing seasons. As in A32, summer classes occur less often (2–4 vs. 6–9 months) than the winter and transitional season classes. Overall, however, A1200 classes are confined to fewer months due to the increase from 32 to 1200 classes. As stated earlier, less averaging occurs for the larger SOM, and classified states extend across a greater range of geopotential heights. Although this field was normalized for the year to contribute evenly compared with other variables, geopotential heights have a distinct annual cycle in response to the varying thermal thickness of the atmosphere. More classes allow for smaller errors in this field and for classes to occur closer to the climatologically expected values.

## SOM results

### General error characteristics

To determine how well a trained SOM represents classified data, SOM_PAK provides the mean quantized error. This unitless quantity is the mean Euclidean distance (*d*) between all classified training samples (*s*) to the class (*c*) within the SOM with the best fit (smallest error) for each sample. For this study, *n* equaled 833 for the number of individual elements in these vectors (Eq. 1), and the number of samples (Nsamp) varied from ~3000 to ~40,000 depending on whether the SOM was annual or monthly.

1 $$ \overline{d}=\frac{{\displaystyle {\sum}_{i=0}^{\mathrm{Nsamp}}\sqrt{{\left({s}_{i1}-{c}_1\right)}^2+{\left({s}_{i2}-{c}_2\right)}^2+\cdots {\left({s}_{in}-{c}_n\right)}^2}}}{\mathrm{Nsamp}} $$

**Fig. 5:**
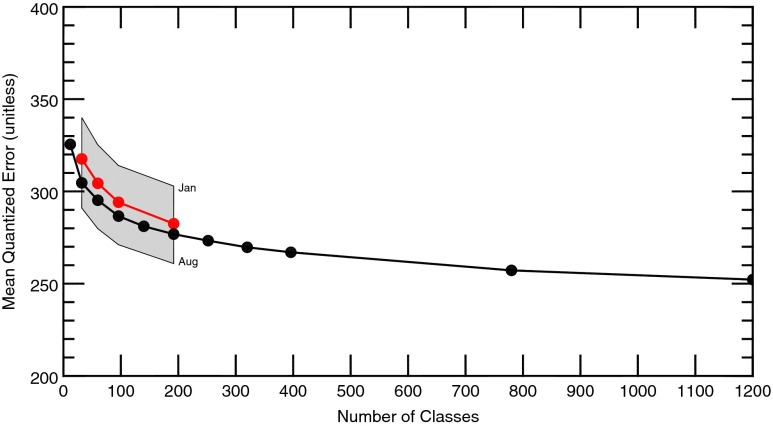
Mean quantized error for annual (*black*) and monthly (*red*) SOMs. The range of values for the 12 months is denoted by the *shaded grey area*

As the number of classes increases, error decreases logarithmically for both annual and monthly SOMs (Fig. 5). The logarithmic profile can be attributed to the use of the neighborhood function that allows the SOM to span the range of data rather than trying to exactly fit it. If error is broken down to individual variables (i.e., the summation in Eq. 1 is only performed for select elements), similar functions are found; however, error is greatest for RH, suggesting that larger variability exists in this field. Error varies by season, with larger errors found during the winter months and smaller errors during the summer (shaded area in Fig. 5). This seasonal variability can be attributed to more quiescent conditions during the summer. During this time of the year in Oklahoma, atmospheric properties in the upper levels are more likely to have weak thermal gradients, allowing for weak or more uniform winds and a compression in the range of potential values for any given field.

### Ability of SOMs to represent climatological CF

SOMs were tested in several ways to determine their ability to fill in time periods of missing observations. In summary, it was assumed that given purely SOM values (only the frequency of classes and class mean CFs are considered), the SOMs should reproduce

- The climatological CF
- Annual cycle of CF
- Month-to-month variability of CF for months with >95 % instrument uptime

Climatological values of CF for all SOMs are within 0.5 % of the observed values (55 % for MMCR + MPL, 45.7 % for MMCR) given in Kennedy et al. (2014). This is expected given relatively even sampling of observations throughout the year; the SOM is effectively acting as a middleman averaging technique, causing samples to first be averaged by class prior to calculating the final average.

**Fig. 6:**
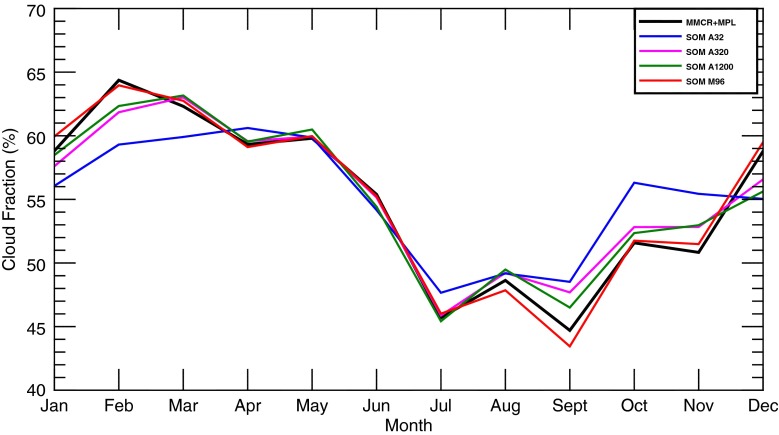
Monthly averaged total CF for select SOMs assuming 0 % instrument availability of MMCR + MPL observations (*colored lines*). For comparison purposes, the observed values from Kennedy et al. (2014) are given by the *black line*

Despite this agreement, SOM performance varies for the annual cycle of CF (Fig. 6). As the number of classes increases for annual SOMs, the ability of the SOM to reproduce the annual cycle increases. SOMs with fewer classes suffer from too much averaging, causing deficits of cloud during the winter and positive biases during the summer and fall. Correlations with observations increase with classes (A32 0.89 → 0.98 A1200) while root mean square errors (RMSEs) decrease (A32 3.14 → 1.44 A1200). Even with 1200 classes, several months have errors on the order of 2–3 %, and the performance increase over 320 classes is marginal at best. Monthly SOMs such as M96 have the best agreement with the annual cycle (Fig. 6). Although not shown, M32 and M60 are in close agreement with M96, with correlations from 0.995 to 0.998 and RMSEs from 0.45 to 0.66. Compared to the annual SOMs, performance of the monthly SOMs is less dependent on the number of classes chosen, although it should be pointed out that from M32 to M96, the effective number of classes increases from 384 to 1152, similar to the jump from A320 to A1200. Potential reasons for superior monthly SOM performance will be discussed later.

To judge month-to-month variability, SOM-derived monthly CFs are compared to observations for months in which instrument uptime was greater than 95 %. For the 14-year (168-month) period, this criterion left 81 (44) months available based on MMCR (MMCR + MPL) availability (Kennedy et al. 2014). Correlations and RMSEs for select SOMs are provided in Table 2. Regardless of the SOM and instrument selection, correlations are high (>0.77), although correlations are slightly higher for MMCR observations. This is largely a function of the nearly double sample size (81 vs. 44 months), allowing for more variability in monthly CFs. Regardless of the type of SOM (annual or monthly), correlations increase and RMSEs decrease as the number of classes increases. Comparisons of the correlations find marginal differences between annual and monthly SOMs, with identical values for M96 and A1200. RMSEs are slightly lower for monthly SOMs, with values 0.22–0.27 % less for M96 vs. A1200. The combination of low RMSEs and high correlations suggests that SOMs can be used to improve instrument observations.

**Table 2:** Correlations and RMSE of monthly CFs for select SOMs to MMCR + MPL and MMCR observations when monthly uptimes are >95 %

	MMCR + MPL		MMCR	
SOM	*r*	RMSE	*r*	RMSE
M96	0.90	4.94	0.93	4.71
M60	0.87	5.42	0.91	5.21
M32	0.84	6.09	0.88	6.08
A1200	0.90	5.21	0.93	4.93
A320	0.83	6.31	0.89	5.71
A32	0.77	7.30	0.86	6.99

### Improving monthly calculations of CF

Given the encouraging results of the prior section, the SOMs are used to fill in instrument gaps. Using the classified atmospheric patterns, periods of instrument downtime are filled with the class mean CFs. To determine how this impacts the uncertainty of monthly calculations of CF, bootstrapping is employed following the methodology of Kennedy et al. (2014). In short, months with uptimes >95 % are selected for bootstrapping. While Kennedy et al. (2014) determined the confidence intervals by randomly withholding data, this study replaces these times with the SOM CF record. The result of this process yields CF errors at the 95 % confidence level as a function of monthly instrument availability. For the sake of completeness, this process is also conducted for the case when instrument downtime is filled with the climatologically expected CF.

**Fig. 7:**
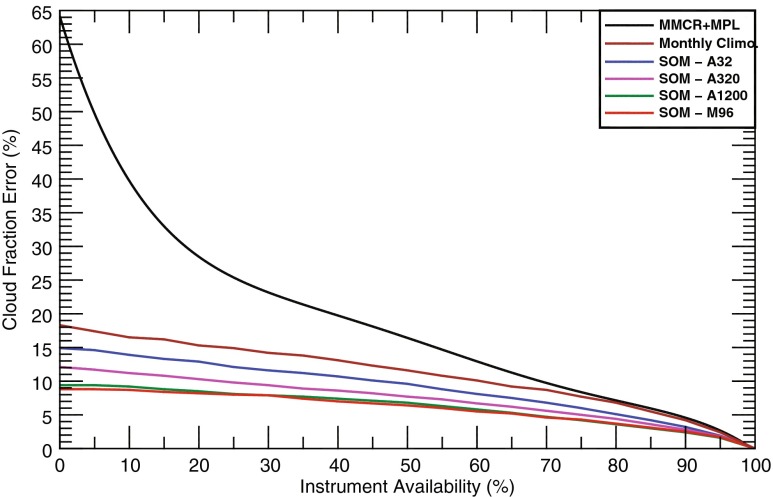
95 % confidence error for monthly MMCR + MPL observations filled with either climatological or select SOM-derived values (*colored lines*). Observed information from Kennedy et al. (2014) is denoted by the *black line*

Inclusion of SOM-derived CFs makes drastic improvements over the original results in Kennedy et al. (2014) (Fig. 7). Whereas the lack of observational data (*black line*) causes errors to rapidly rise for lower instrument availabilities, the inclusion of SOM knowledge yields functions that are near asymptotic. The 95 % confidence error decreases as the number of classes increases, and the larger SOMs (M96 and A1200) have values of 9.1 and 8.8 %, respectively, for months with 0 % instrument availability. More importantly, these SOMs yield errors significantly lower than monthly climatology (~18 % at 0 % availability). In addition, climatology yielded no significant improvements for availabilities greater than 75 %. Even simple SOMs such as A32 yield noticeable gains compared to the former study. In conclusion, any of the SOMs can be used to make significant improvements to uncertainty for the MMCR and MMCR + MPL instrument records at the ARM SGP site.

### Discussion

As the previous two sections have shown, there are notable gains by using SOMs with a higher number of classes. Further, monthly SOMs such as M96 edge out the performance of the annual counterparts such as A1200. Although some statistics are nearly identical (i.e., correlations for monthly CF, see Table 2), monthly SOMs had lower RMSEs and better agreement for the annual cycle of CF (Fig. 6) and yielded marginally better performance for confidence intervals (Fig. 7). The more manageable number of classes for monthly SOMs also has the additional advantage of making visual inspection of the feature map a possible endeavor.

One important question that must be asked is why do monthly SOMs with a similar effective number of classes outperform annual SOMs? Whereas monthly SOMs have a fixed number of classes to draw upon for each month, the A1200 SOM samples from hundreds of classes to determine monthly CFs. This large increase in classes suggests sampling may have a large influence on the calculation of monthly CFs. While monthly and annual SOMs have near-Gaussian probability distribution functions (PDFs) of samplers per class, segregation by month yields PDFs for annual SOMs that are one-tailed and have a maximum closer to 0. In other words, annual SOMs are more reliant on CF information from other months (i.e., July CF is dependent on atmospheric states that occur in other months such as August, June, etc.).

This leads to the next question: why should sampling matter? Stratification of results by month is a purely human endeavor as the atmosphere does not care whether a specific pattern occurs in one month or another. What does change throughout the year, however, are characteristics of the reanalysis used for classifying atmospheric states. As shown in Kennedy et al. (2011), NARR has biases in atmospheric state variables when compared to observed soundings. Further, biases in fields such as RH have seasonal variability. Given the strong correlation of CF with RH found for the SOMs, any seasonally dependent biases will impact performance. For this reason, large biases (yet strong correlations) in CF are found when classifying atmospheric states in one reanalysis using SOMs trained from another reanalysis (not shown).

## A revised record of monthly CF

**Fig. 8:**
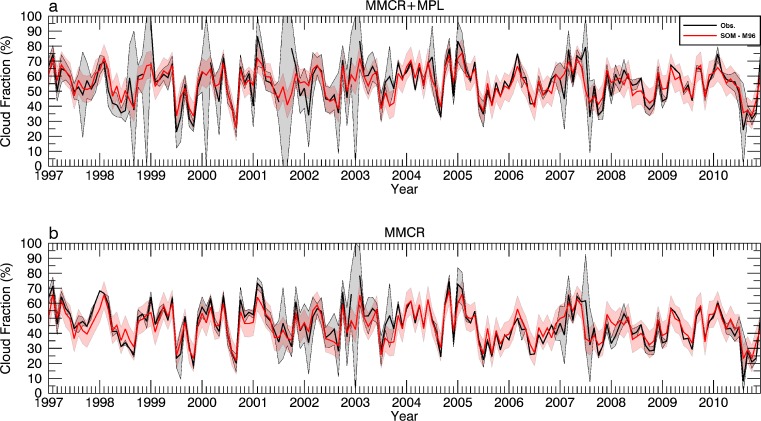
Monthly records of CF from 1997 to 2010 for the M96 SOM (*red line*) along with the 95 % confidence interval (*shaded red*) assuming 0 % availability for MMCR + MPL (**a**) and MMCR (**b**) observations. Observed values and confidence intervals from Kennedy et al. (2014) are denoted by the *black lines and shaded grey areas*

For the reasons outlined above, a revised record of CF at the ARM SGP site is calculated using information provided by the M96 SOM. For comparative purposes, the full 14-year period of monthly CFs for MMCR and MMCR + MPL observations from Kennedy et al. (2014) is provided in Fig. 8. Plotted along with this information is the synthetic record of CF from the M96 SOM assuming 0 % instrument availability. Even for this extreme case, there is excellent agreement between the two records, with significant overlap throughout the time series, backing up the RMSE and correlation values given in Table 2. This agreement suggests that it might be possible to extend the CF record back in time prior to the existence of the ARM SGP site. Prior to this endeavor, however, concerns with shifting atmospheric states with time (climate change and decadal variability) need to be adequately addressed.

**Fig. 9:**
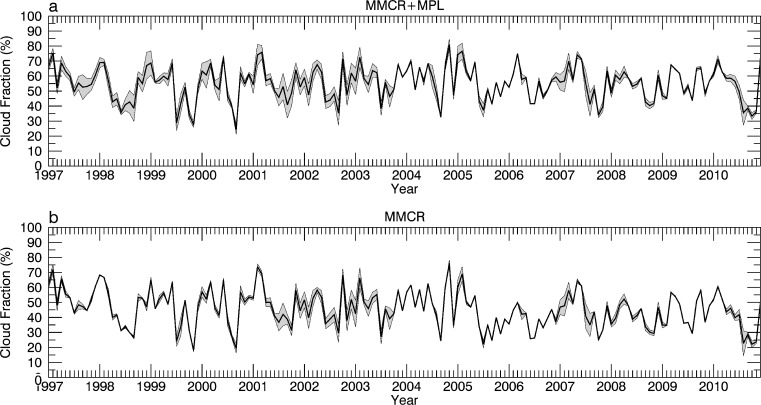
Monthly records of CF from 1997 to 2010 using observations filled with information from the M96 SOM. 95 % confidence intervals are *shaded in grey*

Using a fifth-order polynomial fit to M96 data in Fig. 7, CF errors at the 95 % confidence level are calculated for the observed availabilities. Using this knowledge, instrument records are supplemented to produce the best estimate record of monthly CFs (Fig. 9). The significant periods of uncertainty found in Kennedy et al. (2014) during the first half of the MMCR + MPL record (1997–2013) are largely eliminated (Fig. 1). Months that had no availability now have CFs within a 95 % confidence level ±8.8 %. In summary, the average CF error at the 95 % confidence level has dropped from 4.93 to 2.12 % for the MMCR and from 9.05 to 3.30 % for the MMCR + MPL records. Instrument downtime issues have now largely been mitigated at the ARM SGP site which should aid future model comparisons and trend analyses.

**Fig. 10:**
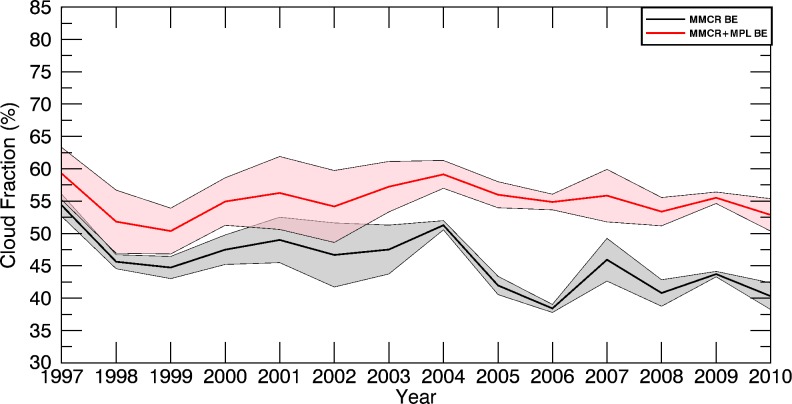
Best-estimate annual total CF from the MMCR (*black*) and MMCR + MPL (*red*) observations filled with information from the M96 SOM. 95 % confidence intervals are given by the *grey and pink shading*

The best estimate of annually averaged total CF is provided in Fig. 10 by combining the monthly results in Fig. 9. Compared to that of Kennedy et al. (2014), overlap between the MMCR + MPL and MMCR instrument records has been almost eliminated. Although no statistically significant trend is present in the MMCR + MPL record, the reduction in uncertainty provides further evidence of the reduction in MMCR CF post upgrades in 2003 (Kennedy et al. 2014).

## Conclusions and future work

In summary, a quantity of SOMs were tested for their ability to classify atmospheric states. This knowledge was then utilized to assess the relationship of classified states to an independently measured variable: total cloud fraction. It is then shown that this information can be used to develop a synthetic record of CF that can either be used independently with excellent agreement to the observed record or to supplement the observed data to significantly decrease uncertainty throughout the 14-year period. While this procedure has demonstrated how SOMs can be utilized to maximize knowledge of the atmosphere and an instrument record to produce a best estimate, this study has also yielded a number of findings relevant to others seeking to utilize SOMs in other studies.

1. Regarding general SOM methodology, the most important aspect of the training process is the number of iterations provided. Sensitivity tests demonstrated that small changes in the neighborhood radius and learning rate had minimal impact assuming an adequate number of iterations are made.
2. Due to the neighborhood function, errors decrease logarithmically with the number of classes. Despite seemingly small changes in mean quantized error, worthwhile gains were found in climatological properties of CF using SOMs with a large number of classes. These types of SOMs were required to minimize monthly CF RMSE and return the largest gains in the reduction of CF uncertainty.
3. Reproduction of the annual cycle of CF requires SOMs to be conducted monthly rather than annually. Monthly SOMs outperformed annual counterparts by various extents throughout this study. The current evidence suggests that this is caused by sampling along with seasonally dependent biases in atmospheric properties within NARR. The high correlations of CF with RH make this field particularly important, and users must be wary of classifying atmospheric states across multiple datasets (i.e., reanalyses and models). As a result, the M96 SOM yielded the best results for this study.

The results of this study pave the way for a number of additional opportunities. Further sensitivity tests are needed to understand how seasonal biases in reanalyses might be mitigated. Another potential avenue of research is using a hierarchal approach for SOMs. In doing so, it may be possible to significantly reduce the number of classes to adequately describe the occurrence of cloud fraction. Cloud fraction in itself is a simplistic depiction of cloud occurrence and radiative properties of the atmosphere. Ideally, the methodology presented in Kennedy et al. (2014) and this study should be extended to classify specific cloud types. In doing so, more worthwhile evaluations of cloud properties in models will be possible. More importantly, the amount of data required to conduct such an analysis needs to be explored to prevent issues with sampling too few atmospheric states. The ARM SGP site will provide a perfect location to determine how long of a record is needed to produce worthwhile results. As Fig. 8 shows, the potential exists to produce “tuned” records of cloud occurrence back in time prior to the existence of observation sites. Such records will be vital for GCM evaluations and investigation of past and current climate trends.

Finally, the developed techniques should be applied to other locations across the globe. Different climates and aspects of the observational record will require further testing of SOMs. This has already been explored to some extent by Stuart et al. (2013) who found fewer classes were needed to describe conditions at the ARM Darwin site.
